# Multichannel Boron Doped Nanocrystalline Diamond Ultramicroelectrode Arrays: Design, Fabrication and Characterization

**DOI:** 10.3390/s120607669

**Published:** 2012-06-07

**Authors:** Raphael Kiran, Lionel Rousseau, Gaëlle Lissorgues, Emmanuel Scorsone, Alexandre Bongrain, Blaise Yvert, Serge Picaud, Pascal Mailley, Philippe Bergonzo

**Affiliations:** 1 CEA-LIST, Diamond Sensors Laboratory, Gif-sur-Yvette 91191, France; E-Mails: emmanuel.scorsone@cea.fr (E.S.); philippe.bergonzo@cea.fr (P.B.); 2 Université Paris Est, ESYCOM EA2552, ESIEE Cité Descartes, BP99, Noisy Le Grand 93162, France; E-Mails: l.rousseau@esiee.fr (L.R.); g.lissorgues@esiee.fr (G.L.); a.bongrain@esiee.fr (A.B.); 3 Institut des Neurosciences Cognitives et Intégratives d'Aquitaine (INCIA), Université de Bordeaux, UMR 5287, Bordeaux 33000, France; E-Mail: blaise.yvert@u-bordeaux1.fr; 4 INSERM, U968, Institut de la Vision, Paris 75012, France; E-Mail: serge.picaud@inserm.fr; 5 UPMC, Institut de la Vision, Université Paris 06, UMR_S968, Paris 75012, France; 6 CEA-LITEN-DTS, Laboratoire de Stockage de l'Electricité, Le Bourget du Lac 73377, France; E-Mail: pascal.mailley@cea.fr

**Keywords:** ultramicroelectrode, boron doped diamond, electrochemical impedance spectroscopy, biosensor array, statistical study of microelectrode arrays

## Abstract

We report on the fabrication and characterization of an 8 × 8 multichannel Boron Doped Diamond (BDD) ultramicro-electrode array (UMEA). The device combines both the assets of microelectrodes, resulting from conditions in mass transport from the bulk solution toward the electrode, and of BDD's remarkable intrinsic electrochemical properties. The UMEAs were fabricated using an original approach relying on the selective growth of diamond over pre-processed 4 inches silicon substrates. The prepared UMEAs were characterized by cyclic voltammetry (CV) and electrochemical impedance spectroscopy (EIS). The results demonstrated that the electrodes have exhibited a very fast electrode transfer rate (k_0_) up to 0.05 cm·s^−1^ (in a fast redox couple) and on average, a steady state limiting current (in a 0.5 M potassium chloride aqueous solution containing 1 mM Fe(CN)_6_^4−^ ion at 100 mV·s^−1^) of 1.8 nA. The UMEAs are targeted for electrophysiological as well as analytical applications.

## 1. Introduction

Microelectrodes exhibit significant advantages over macro-electrode systems, such as a decreased ohmic drop, hemispherical diffusion layer, which extends into the solution, fast establishment of a steady-state signal, and higher S/N ratio. Moreover, they require very small sample volumes [1]. Several multi-electrode arrays systems have been reported elsewhere, where all microelectrodes are connected together to form one single probe [2,3]. Those electrodes, when designed appropriately, still behave as individual microelectrodes at suitable scan rates, but since they are all interconnected they offer other advantages such as an enhanced electrical signal when compared to single electrodes. On the other hand, boron doped diamond (BDD) materials exhibit superior electrochemical properties over other conventional electrode materials including low capacitive background currents, wide potential window in aqueous media and corrosion resistance in harsh environments [4–6]. Thus the advantage of using MEAs over macro-electrodes has been further improved by combining their unique properties resulting from geometrical characteristics with the excellent electrochemical properties of BDD materials [7–10]. Diamond MEAs were used for electro-analytical applications such as, for example, the detection of catecholamine [11]. BDD MEAs were also used to study the formation of different species at the electrode surface, investigated by scanning electrochemical microscopy, such as the peroxodisulfate [12]. To enable the patterned growth of diamond, we had anticipated the approach that was to be used and reported it in reference [13]. However, a step-by-step optimization of the fabrication process has appeared necessary to enable the electrodes to offer the required performances. Here, we have now a clearer view of the best passivation layer that was compatible with the entire process and that offer the required electrode performances. Further we believe our process to be more likely to be compatible with standard fab plants as it avoids diamond etching steps often seen as a limitation for process transfer due to reactor contamination issues.

Several other approaches to fabricate interconnected and individually addressable diamond MEAs were previously described in the literature: Hess *et al.* have reported on fabrication and characterization of a 10 channel diamond on polymer MEA [14], Gao *et al.* on a four channel MEA to detect catecholamine [15]. However for electrophysiological applications, much more miniaturized electrodes are desirable to facilitate more channel of communication. To the authors' knowledge there is no report on fabrication and characterization of BDD micro-electrode arrays (MEA) and ultra-MEA (UMEA) for systems consisting of an array of 64 multiple electrodes or more, where each electrode is individually connected to a single electrical connector and towards a multichannel readout system. Such systems appear extremely useful for electrophysiology applications, where they are used to record the neural signals simultaneously on a 2D network of a cell line culture, a neural tissue or an embryonic organ [16]. Noble metals like platinum [17] and gold [18], as well as silicon [19] microelectrodes have a long history as neural electrodes for electro-analytical systems, but suffer somehow from long term stability. Wang *et al.* have also reported on functionalized hydrophilic carbon nanotube (CNT) microelectrode arrays as a novel prototype neural interface owing to their high charge injection limit [20]. However, some groups have reported on the cytotoxicity of CNTs and hence the biocompatibility issue, especially during long term *in-vitro* measurements, remains an issue to address [21]. Titanium nitride and iridium oxide microelectrodes are extensively used for electrophysiological applications for their enhanced charge injection limit [22]. Although these materials possess high capacitance, the reduced potential window and high background current limit their use in electroanalytical applications. BDD, because of its unique properties appears as a promising alternative material for individually addressed MEAs, and in particular its bio-inertness [6], corrosion resistance and long term stability offer novel interests for electrophysiology applications. Moreover, the electrochemical intrinsic properties of BDD as well as its carbon nature enables covalent immobilization of functional groups such as enzymes, DNA, *etc.* onto the electrode surface [23], further adding highly valuable properties for analytical applications, with the possibility to e.g., selectively anchor antibodies to localize cells. Non-invasiveness, long term and multichannel recording ability makes BDD UMEA the ideal tool for *in-vitro* pharmacology drug research. For example, *in-vitro* electrophysiological response of neural tissues, upon contact with toxic compounds, can be studied using MEA [16]. Another attractive feature of UMEAs is that they can be employed to detect some electroactive species at low concentration and even in the absence of a supporting electrolyte [1,24] since in this particular case the ohmic drop issues get minimized because of the small current range used in such UMEA systems.

Various approaches have been reported in the literature to fabricate BDD MEA and are generally based on standard photolithography techniques along with selective Reactive Ion Etching (RIE) etching of diamond coating [25–27]. We report herein a different technological process for the fabrication of diamond UMEAs, which involves the selective growth of diamond over silicon substrates, followed by the deposition of metal contacts and passivation layers. Microelectrodes exhibit sigmoidal voltammetric curves rather than the peak behavior as observed on macro-electrodes. This sigmoidal behavior is due to steady-state diffusion of species from the liquid to the very small area electrode, whereas on macro-electrodes, the peak behavior is associated with the linear diffusion-limited transport of analytes. The smaller the surface area of the electrode, the better the mass transport towards the active area because of hemispherical diffusion [28–30]. Here we have used electrochemical (EC) characterization techniques such as cyclic voltammetry (CV) and electrochemical impedance spectroscopy (EIS) to appreciate the electrodes performances namely their limiting currents (i_lim_), electron transfer rates (k_0_), electrochemical windows and background currents (i_BG_) *etc.* They are the most efficient techniques to detect any cracks or discontinuity of the insulating layer and analyzing EC properties of individual BDD UMEs.

## 2. Experimental Section

### 2.1. UMEA Fabrication

**Figure 1.**
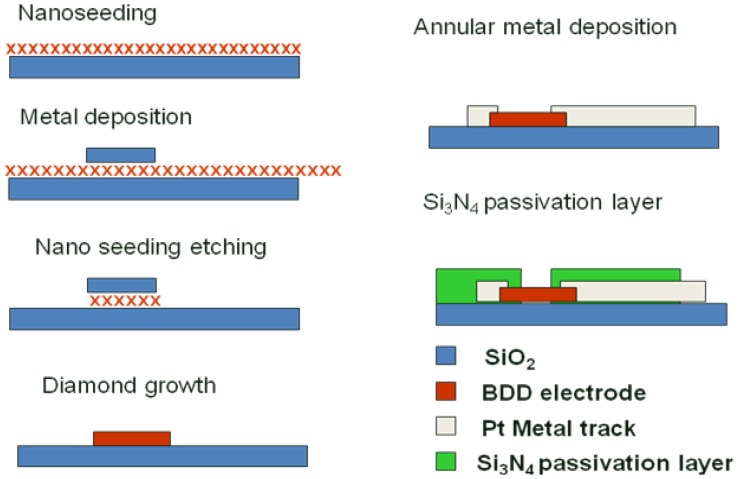
Schematics of diamond ultramicroelectrode array fabrication process.

A process was optimized to allow the fabrication of 8 × 8 diamond UMEAs with diamond electrodes of 14 μm in diameter and 100 μm of inter-electrode pitch. At first, detonation diamond nanoparticles were spread onto a pre-oxidized 4 inches silicon wafer using a process described by Scorsone *et al.* [31]. Next, an aluminum hard mask, composed of 8 by 8 plots of 40 micrometer in diameter each, was deposited over the electrode areas by photolithography and the diamond nanoparticles outside these protected areas were etched away using ultra short oxygen plasma. The metal hard mask was then chemically removed to reveal the diamond nanoparticles patterns, on which diamond electrodes were grown in an MPECVD diamond growth reactor (Seki AX6500) with a gas mixture of methane, hydrogen and trimethylboron at typically 800 °C. The fabricated diamond electrodes exhibit a thickness of approximately 300 nm over 40 micrometers in diameter. The electrodes were then individually contacted by the deposition of Ti (50 nm)/Pt (150 nm) metal tracks using the lift-off process and Clariant Nlof 2020 as photoresist material. Contact on the electrodes was achieved by deposition of a metal ring across the edges of the electrodes. Finally a 600 nm thick silicon nitride (Si_3_N_4_) layer was deposited by CVD onto the substrate in order to isolate the metal tracks from the electrolyte solution. Several insulating passivation layers were tested, including photo-resist SU-8, silicon oxide *etc.*, but leakage capacitive and/or faradic current were observed. Si_3_N_4_, having almost twice the dielectric constant of SiO_2_, was chosen as the passivation layer because of lower leakage current and high resistance to oxidation [32]. The thickness of passivation layer was chosen based on trial and error in order to minimize the leakage current. Openings of the contact areas and of the diamond electrodes were achieved using local etching of the Si_3_N_4_ layer by RIE with SF_6_ gas. It is this process step that determined the final diameter of each individual diamond electrode forming the UMEA. Finally the photoresist used to selectively etch the nitride layer was removed and the UMEA was diced using a diamond saw. The detailed fabrication process is depicted in Figure 1.

Several UMEAs with diameters varying from 2 μm to 25 μm have been fabricated using this technique. Most of these electrodes exhibited typical BDD responses. The complete EC characterization of one such UMEA (with 14 μm diameter) is discussed in detail.

### 2.2. Apparatus for EC Characterization

All the EC characterizations were carried out using an Autolab PGSTAT 302 potentiostat. A three electrode setup consisting of the BDD UME as working electrode, a platinum wire mesh as counter electrode and an Ag/AgCl (3 M KCl) reference electrode was employed in the experiments. For EIS, the Ag/AgCl electrode was replaced by a Pt pseudo-reference. EIS was recorded over a frequency range of 50 kHz–0.1 Hz with logarithmic point spacing and potential amplitude of 0.01 V·rms, while the BDD electrode was maintained at open circuit potential. k_0_ was determined from the Nyquist plot fitted using the ZSimWin 3.21 software.

### 2.3. Reagents

Ultrapure deionised (DI) water (Millipore Direct Q3) was used for all solutions. EIS was performed in an aqueous solution containing 0.5 M potassium chloride (Acros Organics) and 1 mM of potassium ferricyanide(III) and of potassium hexacyanoferrate(II) trihydrate (both from Acros Organics). 0.5 M lithium perchlorate (Sigma Aldrich) aqueous solution was used as the electrolyte for the measurement of the potential window. The steady state limiting current plateau was observed in a 0.5 M potassium chloride aqueous solution containing 1 mM Fe(CN)_6_^4−^ ion while the electrodes were scanned at 100 mV·s^−1^ from 0.05 V to 0.45 V *vs.* Ag/AgCl. The electrode electron transfer rate k_0_ is defined as [33]:

(1) $${\text{k}}_{0}=\text{RT}/{\text{n}}^{2}{\text{F}}^{2}{\text{SR}}_{\text{T}}{\text{C}}_{0}$$

where R is the universal gas constant, T the absolute temperature (K), S the surface area of the electrode (cm^2^), F Faraday's constant (96,500 C mol^−1^), R_T_ the electron transfer resistance of the electrode (ohm), C_0_ the concentration of redox couple (mol cm^−3^), and n the number of electrons transferred. The limiting current i_lim_ is given by the following equation [34]:

(2) $${\text{i}}_{\lim}=4\text{nrFDC}$$

where n is the number of electrons transferred, r the radius of the electrode, D the diffusion coefficient of Fe(CN)_6_^4−^, and C the bulk concentration of the species. Unless stated otherwise the potential is given versus an Ag/AgCl reference electrode through the paper.

## 3. Results and Discussion

**Figure 2.**
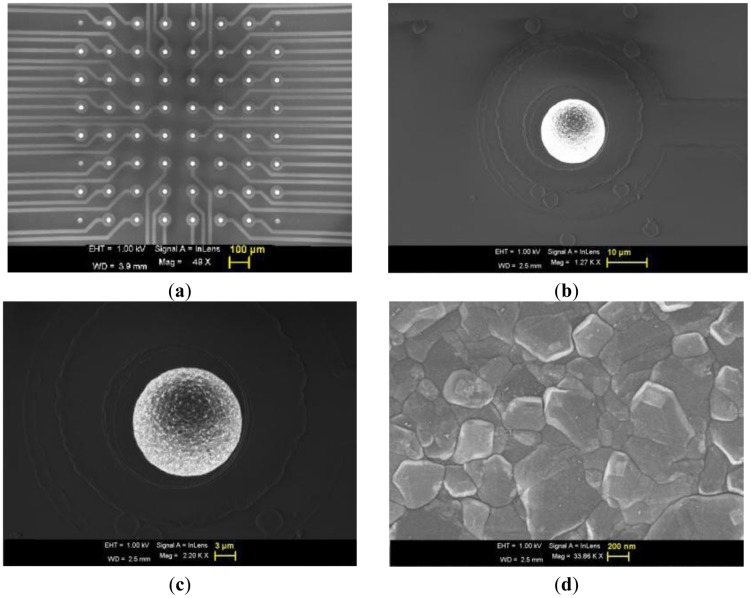
SEM image of an 8 × 8 UMEA along with the tracks (**a**) and magnified SEM image of a single electrode (**b, c** and **d**).

Figure 2(a) shows the SEM image of the 8 × 8 UMEA along with the tracks, where one can observe that each electrode is well separated (100 μm pitch).The diamond crystals are highly faceted, with an average grain size of 100 nm, as seen in Figure 2(d) at higher magnification. Here neither major cracks nor pin-holes were visible, under SEM or optical microscopy, nor on the tracks, nor on the passivation layer nor on the electrode surface as seen in Figure 2(b,c). The electrode surface appears bright in SEM owing to the fact that the surface is conductive in steady state with respect to the outer passivation. In fact the observed surface conductivity may be associated to the hydrogen (H) termination of the electrode surface just after growth. A negative electron affinity is generated on the H terminated surface which increases the density of the back scattered electron [35]. The electron affinity of an H terminated diamond surface can go up to −1.3 eV [35,36].

**Figure 3.**
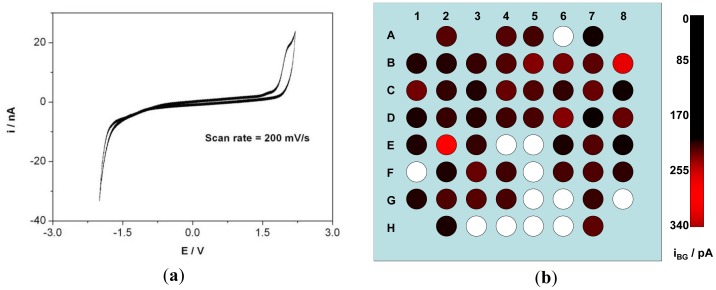
(**a**) Potential window observed by cyclic voltammetry (scan rate = 200 mV·s^−1^) in LiClO_4_ solution where i is the current in nA and E is the applied voltage in volts versus an Ag/AgCl reference electrode and (**b**) RGB model of an 8 × 8 electrode array where each electrode is represented by a spot and the red component value corresponds to their respective i_BG_ value. (In this first prototype, and although measurable, white spots correspond to electrodes that were not exhibiting the EC window of diamond, as associated with Pt shorts from leaky tracks, thus not relevant for the comparison).

**Figure 4.**
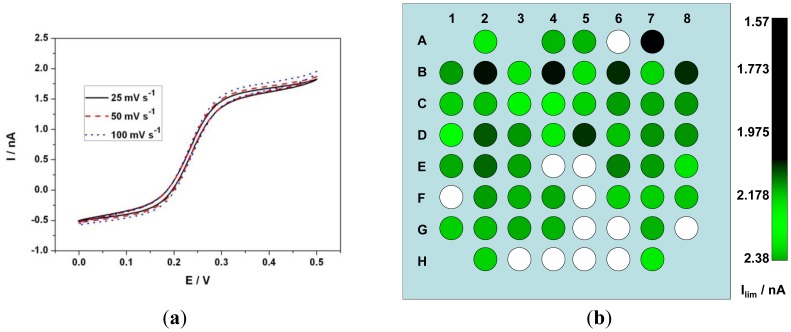
(**a**) Cylcic voltammogram of an electrode at 25, 50 and 100 mV·s^−1^ in 0.5 M potassium chloride aqueous solution containing 1 mM Fe(CN)_6_^4−^ ion and (**b**) RGB model of an 8 × 8 electrode array where each electrode is represented by a circle and the green component value corresponds to their respective i_lim_ value.

**Figure 5.**
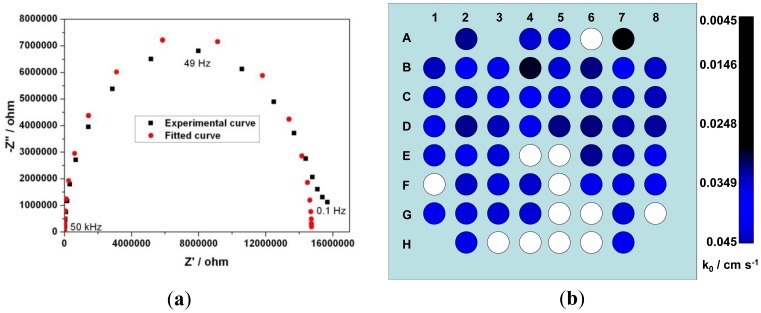
(**a**) Nyquist plot (experimental data and fitted data) of an ultramicroelectrode and (**b**) RGB model of an 8 × 8 electrode array where each electrode is represented by a circle and the blue component value corresponds to their respective k_0_ value.

CV was recorded on all the electrodes in aqueous LiClO_4_ solution to ascertain the accessible EC window. Most of the electrodes exhibit a typical BDD window of over 3 V (in aqueous solution). CV of one such electrode is shown in Figure 3(a) where the electrode was scanned at 200 mV·s^−1^. One unexpected characteristic appeared on this first prototype, as several electrodes exhibited the typical potential window of platinum electrodes. They will be made visible on the following Figures 3(b), 4(b) and 5(b) as white spots, although of course they could be fully characterized but not relevant with the properties of diamond. This was associated with cracks or pinholes in the passivation layer so that the platinum tracks were in contact with the electrolyte. This, although, not observed with imaging techniques becomes easily identified with EC characterization. Other technique to detect the cracks or pinholes is fluorescence confocal laser scanning microscopy as demonstrated by Rudd *et al.* on platinum UMEA [37]. During CV characterization of each electrode, a transient current flows within the potential window when the potential is varied, as the ions move to the surface forming a double layer [38]. The charging and discharging of this double layer constitute to the background current within the potential window. The background current i_BG_ was calculated for the electrodes of the array and was observed to be 94 ± 65 pA at a scan rate of 200 mV·s^−1^. i_BG_ of the 8 × 8 matrix is depicted in Figure 3(b). RGB color model was used to indicate the amplitude of i_BG_ of each electrode. The green and blue color component has a value = 0 and red component vary from 0 to 255 corresponding to the amplitude of i_BG_. The darker the spot, the higher the background current.

Ideally, the limiting current of a UME is independent of the scan rate [39,40]. The radial diffusion behavior of the UME enhances the mass transport and hence shows a sigmoidal behavior in CV. On the other hand a macro-electrode shows a peak and the value of the peak current is directly proportional to the square root of the scan rate as the planar diffusion behavior limits the current. Steady state voltammograms were recorded using CV in 1mM Fe(CN)_6_^4−^ in 0.5 M KCl solution at 100 mV·s^−1^. The limiting current i_lim_ of the 8 × 8 matrix was recorded and the average limiting current value was of 1.83 ± 0.2 nA as opposed to theoretical limiting current of 1.8 nA (Diffusion coefficient of ferrocyanide = 6.67 × 10^−6^ cm^2^·s^−1^ [41]). Furthermore they varied from electrode to electrode with a standard deviation of 190 pA (Table 1). Figure 4(a) shows CV of an electrode at various scan rates. When the diffusion layer d [given by d = (2Dt)^0.5^] is greater than the electrode radius r, radial diffusion predominates and if r ≫ d, planar diffusion predominates, where t is the time of experiment. At slower scan rates of 25, 50 and 100 mV·s^−1^ the diffusion layer thickness d ≫ radius r of the electrode and hence radial diffusion predominates and as a result a sigmoidal voltammogram is observed. i_lim_ observed at 25 mV·s^−1^ was 1.6 nA and at 100 mV·s^−1^ was 1.66 nA. Although a slight deviation from the ideal scan rate independent behavior was observed, the current density variation is negligible (less than 4%) when compared to a macro-electrode at these scan rates. Figure 4(b) represents an RGB color model indicating the value of i_lim_ of each electrode where red and blue color component has a value = 0 and green component vary from 0 to 255 corresponding to the amplitude of i_lim_.

The Nyquist plots obtained from EIS of the UMEA were used to calculate the k_0_ of each electrode. An electrochemical system, represented by Randles circuit, consists of four impedance components: R_S_ the electrolytic resistance, R_T_ the charge transfer resistance, C_D_ the double layer capacitance and Z_W_ the Warburg element. The diameter of the semi-circle portion corresponds to the transfer resistance [42,43]. From the Nyquist plot of one such electrode (Figure 5(a)) it was observed that the straight line (Warburg element) almost disappeared and the plot is more semi-circular. The linear correlation of Re (Z) *vs.* -img (Z) corresponds to a diffusion limited process [44]. The rate of mass transport to and from the electrode is greater in UMEs when compared to macro-electrodes. Hence the Randles circuit of a UME gets modified to a three impedance component (R_S_, R_T_ and C_D_). An R (CR) model circuit was used to fit the experimental curves and the impedance value obtained for R_S_, R_T_ and C_D_ were 414 ohm, 14.7 Mohm and 132 pF respectively. The χ^2^ error was suitably low (χ^2^ < 10^−4^), and the error associated with each element was less than 5%. The electron transfer rate of this electrode was calculated to be 0.012 cm·s^−1^.The semicircle impedance spectra could also favorably be used for bio-sensing applications where one can observe the change in the k_0_ value [42]. The electrode electron transfer rate k_0_ is calculated using Equation (1) and variation of k_0_ along the electrodes in the chip is shown in Figure 5(b). The average value obtained for k_0_ is of 0.013 cm·s^−1^ which is close to the values reported by other groups for an H terminated BDD electrode [45–47]. The electron transfer rate of the electrodes was further enhanced by applying an electrochemical treatment [48]. A train of 50 current pulses of alternating amplitude (10 mA·cm^−2^ and −10 mA·cm^−2^) and of equal duration (100 ms) applied between working and counter electrodes in 0.5 M LiClO_4_ solution. After this treatment the k_0_ values of the electrodes have been augmented by several folds and i_lim_ measured was close to theoretical values.It has been observed that the values of k_0_, i_lim_, C_D_ and i_BG_ were proportional for most of the electrodes. The variation of these values from electrode to electrode cannot be explained by conventional EC and SEM characterization. These variations might be due to difference in intrinsic conductivity of individual grains [49,50]. This is due to differential boron intake, even at higher boron concentration, and hence the electrical and electrochemical characteristics of grains vary from one another. A comparison is made to correlate different parameters such as the k_0_, i_lim_, C_D_ and i_BG_ of 2 electrodes and are summarized in Table 2. The k_0_ and C_D_ values were obtained from nyquist plot and the background current (which is attributed by the double layer capacitance) is higher when C_D_ is higher. The difference in the values of double layer capacitance measured using EIS and CV is 59% and 48% for electrode 1 and 2 respectively. The effective surface area of the electrode depends up on the roughness of the electrode and this variation might also vary the i_lim_ and k_0_ values of the 2 electrodes.

## 4. Conclusions

BDD UMEAs suitable for use in electrochemical sensors were prepared by a microfabrication technique. Topographical characterization and detailed electrochemical study on individual UMEs were carried out. In electrochemical tests, the UMEs exhibited low background currents, almost theoretical steady state limiting currents and fast electron transfer rates (close to 0.01 cm·s^−1^). Further improvement in these two values can be achieved through EC activation. Although several of the UMEs failed to exhibit a BDD response, those can be easily discriminated and the process is currently being improved to completely eliminate such a failure rate. The long term goal of our work is ultimately to develop biosensing platforms for the monitoring of neural activities for electrophysiology.

## Figures and Tables

**Table 1.** Mean value and standard deviation of the background current i_BG_, limiting current i_lim_, and transfer rate k_0_.

**Parameters**	**Mean value**	**Standard deviation**

i_BG_	94.33 pA	64.85 pA
i_lim_	1.83 nA	0.19 nA
k_0_	0.0132 cm·s^−1^	0.008 cm·s^−1^

**Table 2.** Comparison of different parameters such as the electron transfer rate k_0_, limiting current i_lim_, double layer capacitance C_D_ and background current i_BG_ of 2 electrodes.

**Parameters**	**Electrode 1**	**Electrode 2**

k_0_ (cm·s^−1^)	0.012	0.017
i_lim_ (nA)	1.74	1.76
C_D_ (pF)	132	197
i_BG_ at 200 mV·s^−1^(pA)	63	76
